# Application of Layered Coding Genetic Algorithm in Optimization of Unequal Area Production Facilities Layout

**DOI:** 10.1155/2019/3650923

**Published:** 2019-06-19

**Authors:** Shiwang Hou, Haijun Wen, Shunxiao Feng, Hui Wang, Zhibin Li

**Affiliations:** ^1^Department of Mathematics, Brunel University London, London UB8 3PH, UK; ^2^School of Business, Huaihua University, Huaihua Hunan-418000, China; ^3^School of Mechanical Engineering, North University of China, Taiyuan Shanxi-030051, China

## Abstract

Unequal area facilities layout problem (UA-FLP) is an inevitable problem in the process of new construction, reconstruction, and expansion of enterprises. The rationality of the facilities layout has a great influence on the operation performance of the production system. Finding the optimal solution of UA-FLP according to the requirement of production process is the main content of the plant design. The facilities were constrained by given areas and aspect ratio, respectively. By adopting the method of slicing tree, the layout space was divided into multiple regions for each facility. The genetic algorithm was developed by using layered coding to show the slicing process. Considering the production logistics cost as well as the adjacency relations between the facilities, the goal function was established and the optimal solution was obtained by running the proposed algorithm. Finally, the feasibility of the proposed approach was validated by a set of known problems. The comparison results show that it can provide decision support for rapid optimal layout of multifacilities.

## 1. Introduction

UA-FLP was proposed originally by Armour and Buffa in [1], and its objective is to determine the good locations for a given set of departments with different areas on some workshop floor to optimize the material handling cost and/or other objectives. The facility can be small or big according to the level of the facility layout, but it should be a physical entity with some function. There is about 20% to 50% of the processing cost used for material handling, and scientific and reasonable facilities layout can save at least 10% to 30% of the material handling fee [2, 3].

Classic FLPs tend to study equal-area facilities arrangement; i.e., all facilities have the same area and shape. In this case, the facility centroid is fixed and the overall closeness or distance between facilities will not change when switching the location of any two facilities. But, the equal-area hypothesis is impractical, and the facility area is often unequal. So, the centroid of each facility depends on its area and shape and has no regular distribution as equal-area case.

**Figure 1:**
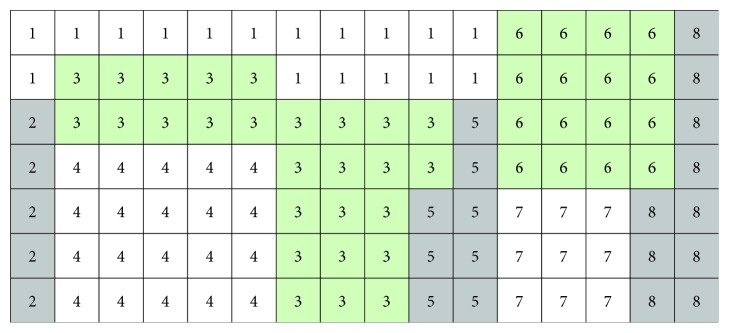
Facility layout diagram of discretization of the planar grid.

Heuristic algorithm based on the discrete model is proposed in reference [4] to deal with the UA-FLP. This approach divided the district into some small squares with a fixed area, and each facility was allocated some numbers of squares most close to its area by use of heuristic algorithm. As shown in Figure 1, the grid size determines the precision of the facility representation; the smaller the grid size, the more precise the facility representation. The facility shape was also represented well by this approach. But, the time consumption is great to calculate the interference between facilities during the detailed layout process. Also, this approach is easy to produce facility layout with irregular shape. By far, the most commonly used UA-FLP model is mainly based on block diagram with unequal area as depicted in [5].

Many researchers have studied the optimization methods for UA-FLP. The literature about the methods can be mainly divided into three categories. The first one is deterministic algorithm to calculate accurate solution, such as mathematical programming [6, 7], mixed-integer linear programming [8], and QAP as aforementioned. The second category is heuristic algorithm mentioned in reference [9–11]. Another category is intelligent algorithm, such as genetic algorithm (GA) [12, 13], particle swarm optimization (PSO) [14, 15], ant colony algorithm (ACA) [16, 17], simulated annealing algorithm (SAA) [18, 19], and tabu search algorithm [20].

Deterministic algorithm has high requirement in memory and CPU time, so it is often used to solve small-scale UA-FLP and reference [21] proved its effectiveness. Heuristic algorithm and intelligent algorithms have faster calculation speed and are suitable for large-scale UA-FLP. Furthermore, genetic algorithm is most widely used, and different genetic algorithms were developed for various UA-FLPs.

A genetic algorithm hybridized with local search to obtain the Pareto solutions set was proposed in [22], and the author adapted a random weight to combine values of two objectives. For unequal area facility layout problems, a genetic algorithm based on slicing structure was developed in [23], and four objective functions such as material handling costs, aspect ratio, closeness, and distance requests were considered simultaneously by use of a Pareto-based evolutionary approach. In order to improve the performance of premature convergence, lack of diversity, and high computational cost, an island model genetic algorithm was proposed in [24]. The compared results showed the proposed approach has great improvement on the above aspects. In [25], a multiobjective interactive genetic algorithm was proposed by considering both quantitative aspects and subjective features, which allowed the interaction between the expert designer and the algorithm. A biased random-key genetic algorithm to determine the placement order and dimensions of each facility was proposed in [26], and the results showed its better performance for 19 of the 28 benchmark facility layout problems.

For large-scale UA-FLP problems, a genetic algorithm combined with a decomposition strategy was proposed in [27]. Compared with basic genetic algorithm, the experiments in the paper showed that the proposed approach had an average solution improvement of 6% or 7% for large-scale instances with 90 or 100 facilities.

This paper put forward a method of LCGA (layered coding genetic algorithm) for slicing-based plane splitting to lay out facilities. This approach can generate feasible solution rapidly with the help of the layered coding method. It can provide larger-scale UA-FLP solving a new thinking. The rest of this paper is organized as follows: Section 2 provides a description of the UA-FLP dealt with in this paper. The design process of the proposed layered coding genetic algorithm is presented in Section 3.  Section 4 compares the proposed approach with some known UA-FLPs. The conclusions are presented in Section 5.

## 2. UA-FLP Description

### 2.1. Location Relations of UA-FLP

**Figure 2:**
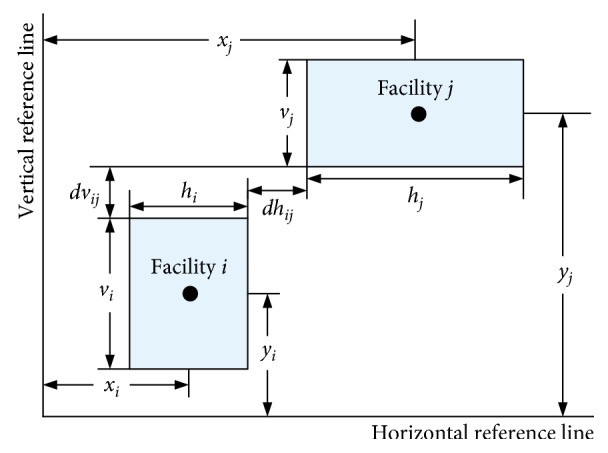
Location relations diagram of the facility layout. *h*_*i*_: horizontal length of facility *i*; *v*_*i*_: vertical width of facility *i*; *x*_*i*_: *x*-coordinate of facility centroid relative to the ordinate origin; *y*_*i*_: *y*-coordinate of facility centroid relative to the ordinate origin; *dh*_*ij*_: the necessary horizontal spacing between facility *i* and facility *j*; *dv*_*ij*_: the necessary vertical spacing between facility *i* and facility *j*.

Assume that all facilities are rectangular blocks with a given area. Considering the building module, facilities need to satisfy a given aspect ratio constraint to avoid producing an approach with long narrow shape facilities layout. All facilities must be located in a given area and cannot overlap between them. Take a UA-FLP involving facilities *i* and *j*; for example, the relative position relations are as shown in Figure 2.

**Figure 3:**
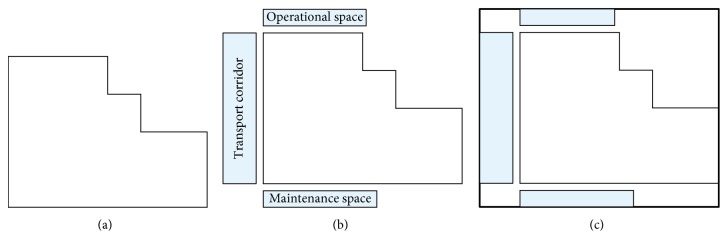
Diagram of facility planar space. (a) Projection polygon of facility space. (b) Projection polygon of facility and operation space. (c) Rectangular envelope of projection polygon of (b).

**Figure 4:**
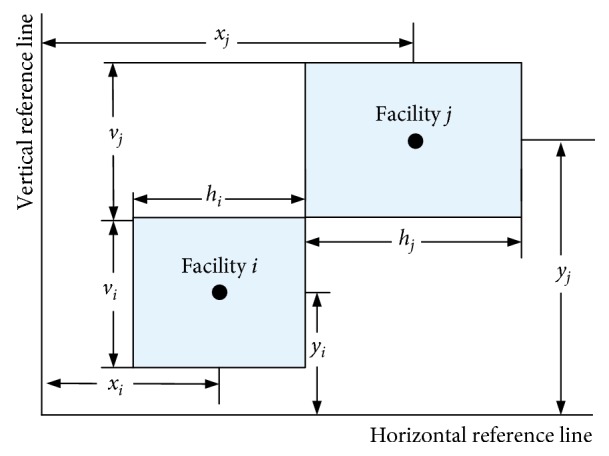
Simplified graphic of the location relation between facilities.

Figure 3(a) shows the projection polygon of facility space.  Figure 3(b) shows the projection polygon of facility and its necessary space, including transport corridor, operational space, and maintenance space for workers. To facilitate the facilities layout, Figure 3(c) considers the rectangular envelope of Figure 3(b) as the objects of UA-FLP. So, the necessary horizontal and vertical spacing between facilities shown in Figure 2 can be decomposed to the corresponding facility (as shown in Figure 4). By doing so, the amount of computation to solve UA-FLP can be decreased without losing the accuracy of result.

### 2.2. Objective Function and Constraints

Satisfying the abovementioned basic hypothesis, the UA-FLP becomes the following optimization problem: arranging *n* facilities to a specified area to minimize the material handling cost and maximize the closeness scores.

#### 2.2.1. Minimizing the Material Handling Cost

(1) $$\begin{array}{c} \text{Min}\;\;G_{1}=\overset{n-1}{\underset{i=1}{\sum }}\overset{n}{\underset{j=i+1}{\sum }}c_{ij}f_{ij}d_{ij}, \end{array}$$

where *n* is the number of facilities, *c*_*ij*_ is the per unit handling cost between facility *i* and facility *j*, *f*_*ij*_ is the logistics quantity between facility *i* and facility *j*, and *d*_*ij*_ is the distance between facility *i* and *j*.

#### 2.2.2. Maximizing the Closeness Scores

(2) $$\begin{array}{c} \text{Max}\;\;G_{2}=\overset{n-1}{\underset{i=1}{\sum }}\overset{n}{\underset{j=i+1}{\sum }}b_{ij}r_{ij}, \end{array}$$

where *n* is the number of facilities and *b*_*ij*_ ∈ [0,1] is the ratio of distance between facility *i* and facility *j* to the maximum distance between facilities in a given layout approach, and is used to represent their closeness factor between two facilities. *r*_*ij*_ is the quantitative score of the nonlogistics relationship level determined by the SLP method (Table 1).

This problem belongs to multiobjective optimization problem (MOOP) since there is more than one objective function to be optimized simultaneously. Optimal decisions need to be taken in the presence of tradeoffs between two or more conflicting objectives. Taking UA-FLP as example, material handling cost is minimized while closeness scores are maximized while locating facilities. For a nontrivial multiobjective optimization problem, no single solution exists that simultaneously optimizes each objective. There exist a (possibly infinite) number of Pareto optimal solutions; i.e., under these solutions, none of the objective functions can be improved in value without degrading some of the other objective values. All these Pareto optimal solutions are considered equally good if there is no additional subjective preference information.

There are many kinds of forms of solutions for different goals, such as a representative set of Pareto optimal solutions and/or a single solution that satisfies the subjective preferences of decision maker. In this paper, we adopt the latter in order to obtain a well-determined layout approach.

Let *M* be the maximum of *r*_*ij*_ and *w*_1_ and *w*_2_ be the weight coefficients of material handling cost and nonlogistics closeness, respectively. The above two goals can be synthesized into a minimizing objective function:

(3) $$\begin{array}{c} \text{Min}\;\;G=w_{1}G_{1}+w_{2}\left(M-G_{2}\right)=w_{1}\overset{n-1}{\underset{i=1}{\sum }}\overset{n}{\underset{j=i+1}{\sum }}c_{ij}f_{ij}d_{ij}+w_{2}\left(M-\overset{n-1}{\underset{i=1}{\sum }}\overset{n}{\underset{j=i+1}{\sum }}b_{ij}r_{ij}\right), \end{array}$$

subject to

(4) $$\begin{array}{c} \frac{UR_{i}\left(x\right)-LL_{i}\left(x\right)}{UR_{i}\left(y\right)-LL_{i}\left(y\right)}\geq \alpha^{\min}, \end{array}$$

(5) $$\begin{array}{c} \frac{UR_{i}\left(x\right)-LL_{i}\left(x\right)}{UR_{i}\left(y\right)-LL_{i}\left(y\right)}\leq \alpha^{\max}, \end{array}$$

(6) $$\begin{array}{c} \frac{UR_{i}\left(x\right)-LL_{i}\left(x\right)}{UR_{i}\left(y\right)-LL_{i}\left(y\right)}=A_{i}, \end{array}$$

where *UR*_*i*_(*x*), *LL*_*i*_(*x*), *LL*_*i*_(*y*), and *UR*_*i*_(*y*) denote the *x*-coordinate and *y*-coordinate of upper-right corner and lower-left corner of facility *i*, respectively. Formulas (4) and (5) ensure the aspect ratio of facility *i* in a given range $\left[\begin{array}{cc} \alpha^{\min} & \alpha^{\max} \end{array}\right]$, and formula (6) constrain the area of facility *i* equal to given value *A*_*i*_.

Manhattan distance is adapted to calculate the distance between facilities, namely:

(7) $$\begin{array}{c} d_{ij}=\left|x_{i}-x_{j}\right|+\left|y_{i}-y_{j}\right|, \end{array}$$

where *x*_*i*_ and *y*_*i*_ denote the *x*-coordinate and *y*-coordinate of the centroid. The exact coordinate position of facility *i*, {*LL*_*i*_(*x*, *y*), *UR*_*i*_(*x*, *y*)}, can be obtained by the following formula:

(8) xi=LLxi+URix−LLxi2,yi=LLyi+URiy−LLyi2.

## 3. Algorithm Design

### 3.1. Basic Layout Principle for UA-FLP Using Slicing Tree

In order to locate all facilities into a given area, the area should be divided into subareas with the same number of facilities. This paper adopted plane segmentation method to generate slicing tree in order to describe the relative position relationships of facilities during locating them.

**Figure 5:**
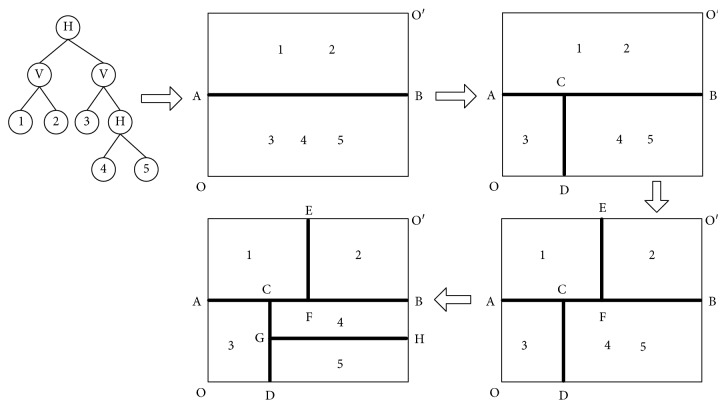
Cutting tree layout process of the plane segmentation method.

For a UA-FLP with *n* facilities, the slicing tree contains *n* leaf nodes and (*n* − 1) internal nodes. The information of the plane segmentation mode is contained in internal nodes, i.e., horizontal split (labeled H) or vertical split (labeled V). Taking a UA-FLP with 5 facilities as an example, a feasible plane segmentation approach is shown in Figure 5.

The process of plane segmenting is the process of facilities layout. The change of coordinates for each partition after each plane segmenting is described as follows. The area has only two points: *O*(0,0) and *O*′(∑*h*_*i*_, ∑*v*_*i*_), when there is no facility located. The coordinate of upper-right corner is marked with the maximum limit value; i.e., *x*-coordinate is the sum of width of all facilities supposing they are side-by-side arranged horizontally, and *y*-coordinate is the sum of length of all facilities supposing they are side-by-side arranged vertically. Taking the first partition as an example, the splitting process is as follows. Suppose AB is the first cutting line. The plane area is divided into two partitions. The upper part is for facilities 1 and 2, and the lower part is for facilities 3 to 5. After the first segmentation, the coordinates of point A and B are *A*(0, ∑_*i*=3,4,5_*v*_*i*_), *B*(∑*h*_*i*_, ∑_*i*=3,4,5_*v*_*i*_).

Similarly, the other four divisions can be done. Finally, all facilities are located in different parts, and the precise coordinates of the facilities are as follows: facility 1{*A*, (*x*_*A*_+*h*_1_, *y*_*A*_+*v*_1_)}, facility 2{*F*, (*x*_*F*_+*h*_2_, *y*_*F*_+*v*_2_)}, facility 3{*O*, (*x*_*O*_+*h*_3_, *y*_*O*_+*v*_3_)}, facility 4{*G*, (*x*_*G*_+*h*_4_, *y*_*G*_+*v*_4_)}, and facility 5{*D*, (*x*_  *D*_+*h*_5_, *y*_  *D*_+*v*_5_)}. So, a feasible layout approach is provided. Based on this layout idea, this paper studies the hierarchical coding genetic algorithm in order to realize the layout scheme iterative optimization.

### 3.2. Genetic Algorithm Design

For a UA-FLP with *n* facilities, there are *n*! possible combinations of their position and the number of combination will be larger if the shape or orientation of each facility is considered. Above all, there is a large number of local optimum in this huge solution space. So, it is a NP-hard problem, and it is advisable to find a suboptimal solution in acceptable cost (money, time, or computing resource) by use of some heuristics search algorithms.

GA is a bionic algorithm for searching the optimal solution based on the principle of biological evolution. It simulates the natural process of gene recombination and evolution and compiles the parameters into binary-code or decimal-code (or other codes) genes. Several genes constitute a chromosome (individual), and many chromosomes carry out operations similar to natural selection, pairing crossover, and mutation. The final optimization result is obtained after repeated iterations (that is, generation inheritance).

#### 3.2.1. Layered Coding Approach

Coding is to map the phenotype data in solution space into genotype data in genetic structure. During iterations of GA, a coding string represents a solution and genetic operations are done by operating the bits of this string. So, the coding method also affects the genetic operators.

There are mainly two coding methods: real number coding and binary coding. The former uses a real number as a gene, is easy to understand, and does not need decoding process, but it is also easy for premature convergence, thus falling into local optimum. The latter uses a binary string with specific length as a gene and has higher stability, larger population diversity, and better performance for global search. In this paper, we adopted the binary coding method. The number of bit is determined by the accuracy of the solution to be achieved.

For example, suppose an *x*-coordinate ranging in [0, 4] and the solution is exactly 4 decimal places behind the decimal point. The solution space is divided into (0-1)^*∗*^ (1e + 4) = 10,000 equal fractions. It takes 14 bits of binary to represent a solution; i.e., the coding of a solution is a 14 bit binary string since 2^13^ < 10000 < 2^14^. The decoding process is as follows:

(9) $$\begin{array}{c} x_{\text{coordinate}}=0+\text{decimal}\left(\text{chromosome}\right)\;*\;\frac{\left(4-0\right)}{2^{14}-1}. \end{array}$$

Generally, for *x* ∈ [lower_bound, upper_bound], the value of *x* after decoding is

(10) $$\begin{array}{c} x=\text{lower\_bound}+\text{decimal}\left(\text{chromosome}\right)\;*\;\frac{\left(\text{upper\_bound}-\text{lower\_bound}\right)}{2^{\text{chromosome\_length}}-1}. \end{array}$$

The coded chromosome string should represent the following information simultaneously: facility sequence for layout, splitting point sequence, and splitting mode. The coding approach will be detailed below.

**Figure 6:**
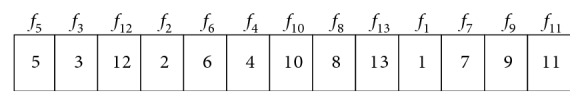
Facility sequence code string of 13 facilities.

Facility sequence code is in the first layer. *N* facilities are coded by *n* different integers in the interval [1, *n*] by use of integer coding. The coding string can be any sequences of *n* integers in the interval [1, *n*] to allow different facility locating orders.  Figure 6 shows a code string [1–13] of 13 facilities, f1,…, f_13_, with a random order of integers from 1 to 13, and the value of each bit of code string denotes the number of the facility it represents.

**Figure 7:**
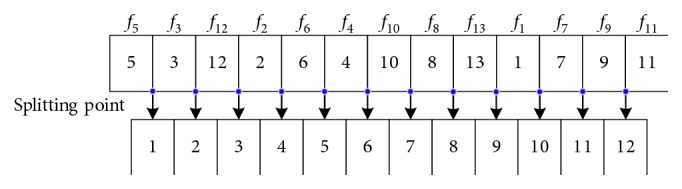
Bit value of splitting point sequence code.

Splitting point code lies in the second layer. A feasible splitting point lies between every two bits of facility coding string. For every two adjacent bits of facilities coding string, there is a splitting point. For a *n*-bit facility coding string, there are *n* − 1 splitting points. We denote every splitting position as an integer between 1 to *n* − 1. For the facility code string in Figure 6, its corresponding splitting position is shown in Figure 7.

**Figure 8:**
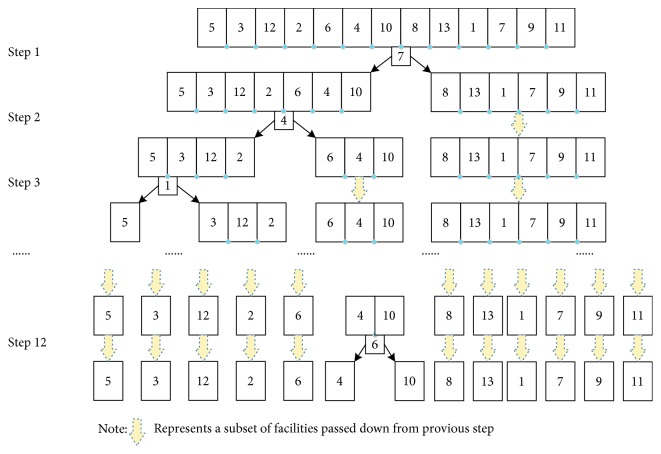
Splitting process according to a given splitting point sequence.

The facility set is divided into two different parts at the splitting point selected firstly. The remainder splitting points are contained in these two subsets of facilities. After each splitting operation, the number of subsets of facilities increases by 1. After *n* − 1 splitting, the facility set with *n* facilities will be divided into *n* single facilities locating in *n* different area blocks. For the code string of splitting point, we code them as a sequence from 1 to (*n* − 1) with random order corresponding to different plane segmentation approaches. The value of each bit of code string denotes the number of the splitting position in the facility code string. Taking the string shown in Figure 6 as an example, there are 12 positions that can be set as splitting points when carrying out plane segmentation. Suppose we produce a splitting point code string as [1–12], the splitting operation will begin with the 7^th^ splitting position, then 4^th^, and finally, 6th. The splitting process is shown in Figure 8.

**Figure 9:**
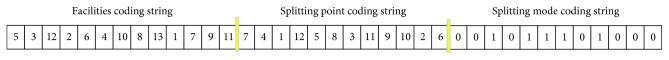
A complete three-layer coding string of UA-FLP with 13 facilities. (a) *n*-bits facilities coding string, (b) (*n* − 1)-bits splitting point coding string, (c) (*n* − 1)-bits splitting mode coding string.

The last layer of coding provides the information of splitting mode, horizontally or vertically. The splitting result of these two ways is different, and the facility layout is also different. We use 0 for horizontal and 1 for vertical. So, the splitting mode code is a binary string that has the same number of bits as the splitting point code. For the example as shown in Figure 8, assume a splitting mode code is [0, 0, 1, 0, 1, 1, 1, 0, 1, 0, 0, 0].  Figure 9 shows a complete three-layer coding string or a chromosome of a feasible solution for a UA-FLP with 13 facilities. For the first step, the splitting mode is horizontal and the process of plane segmentation is similar to the description in Section 3.1.

**Figure 10:**
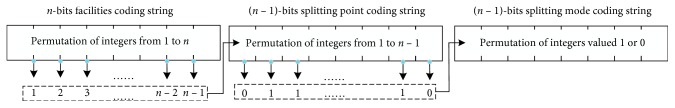
Layered coding chromosome gene string.

So, for a UA-FLP with *n* facilities, the three-layer coding string can be expressed as shown in Figure 10.

#### 3.2.2. Crossover Operation

In genetic algorithms and evolutionary computation, crossover, also called recombination, is a genetic operator used to combine the genetic information of two parents to generate new offspring. It is one way to stochastically generate new solutions from an existing population. Newly generated solutions are typically mutated before being added to the population.

The coding method determines the data structures to store genetic information and also affects the crossover operators.

Due to the aforementioned layered coding structure, genetic operation must be carried out by the segment to ensure the feasibility of the new code string. Taking the chromosome gene string in Figure 7 as an example, crossover points are selected in three layers, respectively. The two parent individuals swap the gene segments before and after the crossover points in three layers, respectively.

**Figure 11:**
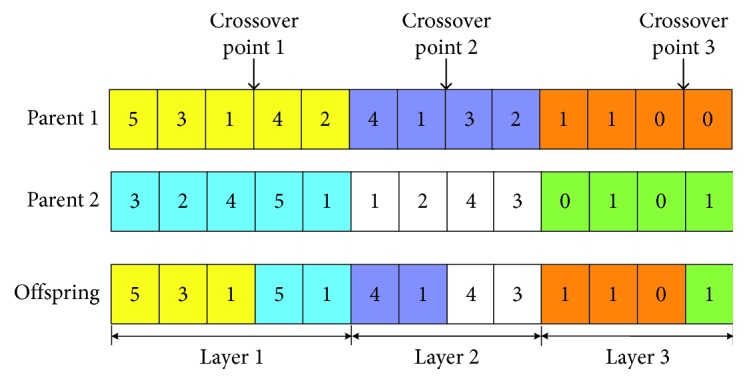
Layered coding crossover operation. (a) Parent 1. (b) Parent 2. (c) Offspring.

A crossover operation of layered coding string of 5 facilities is shown in Figure 11.

It can be found that some bit value of new code strings will lose or repeat in the first two layers after crossover operation. So, repair operation is necessary. Sort the missing value in ascending order and replace the repeated value of corresponding layer code strings. Taking the above offspring as example, the layer 1 code of offspring is [5 3 1 5 1]. The reappeared values are 5 and 1, and the missing values are 2 and 4. We repair the offspring code of layer 1 as [5 3 1 2 4]. After similar repairment, the offspring code of layer 2 is [4 1 2 3].

#### 3.2.3. Mutation

Mutation changes one or more gene values in a chromosome from its initial state in order to maintain genetic diversity from one generation of a population to the next. This can also prevent the population of chromosomes from becoming too similar to each other, thus slowing or even stopping evolution. For the layered code, we also adopted different mutation operators.

A mutation operator involves a probability *P*_m_ that an arbitrary bit in a genetic sequence will mutate from its original state. A common method of implementing the mutation operator involves generating a standard uniform-distributed random number *R* for each bit in a sequence.

The corresponding bit will be modified if *R* > *P*_m_. This single-point mutation is suitable for third-layer binary code of an individual.

For the first- and second-layer code, mutation operation is implemented by interchanging two genes of code string in order to ensure the feasibility of new individual after mutation. The gene bits to mutate are selected randomly and also use a standard uniform-distributed random number *R* to determine whether or not the selected bits will be swapped.

### 3.3. Processing for UA-FLP with Empty Space

The method proposed herein split the area into subareas that are consistent with the number of facilities, each of which accommodates a facility. For a UA-FLP with empty space, we firstly need to turn it into a UA-FLP without empty space. The detailed process is explained as follows.

**Figure 12:**
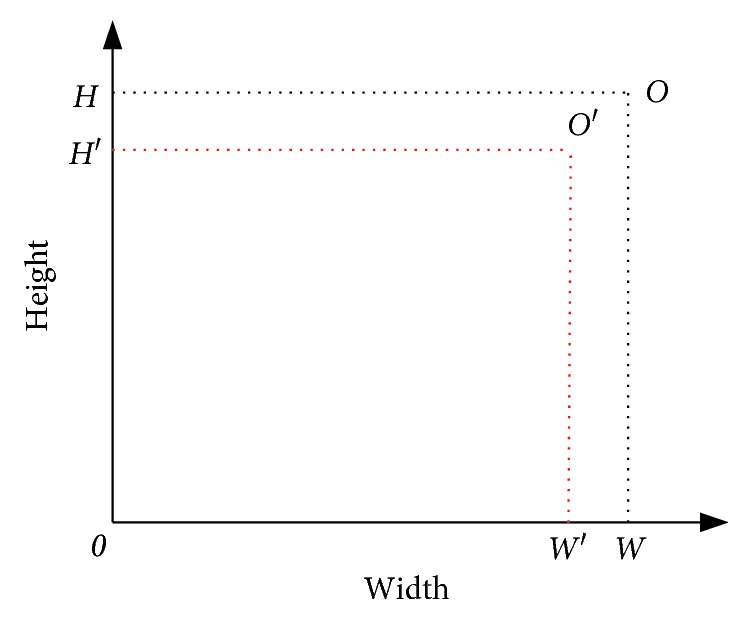
Layout diagram for UA-FLP with empty space.

Suppose the ratio of the original region for all the facilities is *r*=*W*/*H* and the area of *i*-th facility is *A*_*i*_, we can arrange all the facilities in a new region, marked by the red border in Figure 12, with the area equal to the sum of all facilities since all necessary spaces for each facility has been included in its area as illustrated in Figure 3. The width *W*′ and height *H*′ of this new region should satisfy the following equations:

(11) $$\begin{array}{c} \left\{\begin{array}{c} \frac{W}{H}=\frac{W{}^{\prime }}{H{}^{\prime }},\\ W{}^{\prime }\;*\;H{}^{\prime }=\overset{n}{\underset{i=1}{\sum }}A_{i}. \end{array}\right. \end{array}$$

In general, solutions found in this way will be better than solutions found in other methods because the horizontal and vertical coordinates of each facility will be smaller.

## 4. Case Study

In order to validate the proposed approach, a set of problems described in the literature was used in this section. All the tested problems are shown in Table 2.

The algorithm is coded in Matlab 2015b. The computer's configuration running the algorithms was as follows: Intel Core i5-4460 (3.20 GHz), 8 GB RAM, and a Windows 10 operating system.

The algorithm parameters settings for the tested problems are listed in Table 3, and the algorithm proposed in this paper is described in detail in Section 4.1.

### 4.1. Parameters Setting and Pseudocode of Algorithm

1. *Coding Method*. Integer coding was adopted in this case. The number *n* of facilities was assigned to *n_f.* The layout approach and the splitting point sequence were denoted by two interpermutations which took values from [1, *n*] and [1, *n* − 1], respectively. The splitting mode of each splitting operation was represented by a thirteen bits encoded string, and each bit can take value 0, splitting horizontally, or 1, splitting vertically.
2. *Parameters Setting*. See Table 3.
3. *The Generation of Initial Population*. According to the above approach, initial facility population, splitting position string, and splitting mode string can be generated by use of Algorithm 1.

Algorithm 2 carries out the establishment of fitness function and its value calculation.

The crossover operation and the corresponding repair approach of facility level are presented in Algorithm 3.

As for the crossover and repair operation of the splitting point level, the method is similar to the above ideas; just replace the *f_popu* and *n_f* with *so_popu* and (*n_f* − 1). There is no need for repair operation in the splitting mode level, and its crossover operation is the same as the facility level.

The mutation operation of the facility level is shown in Algorithm 4.

The mutation operation of the splitting point level or splitting mode level can be completed by replacing the *f_popu* and *N_f* of Algorithm 4 with *so_popu* and (*n_f* − 1) or *sp_popu* and (*n_f *−* *1).

### 4.2. Computational Results and Analysis

According to the above parameters setting, all the tested problems were solved by the proposed layout algorithm; the average optimal value of objective function of each tested problems was obtained as shown in Table 4 by running the proposed algorithm 30 times for each problems.

**Figure 13:**
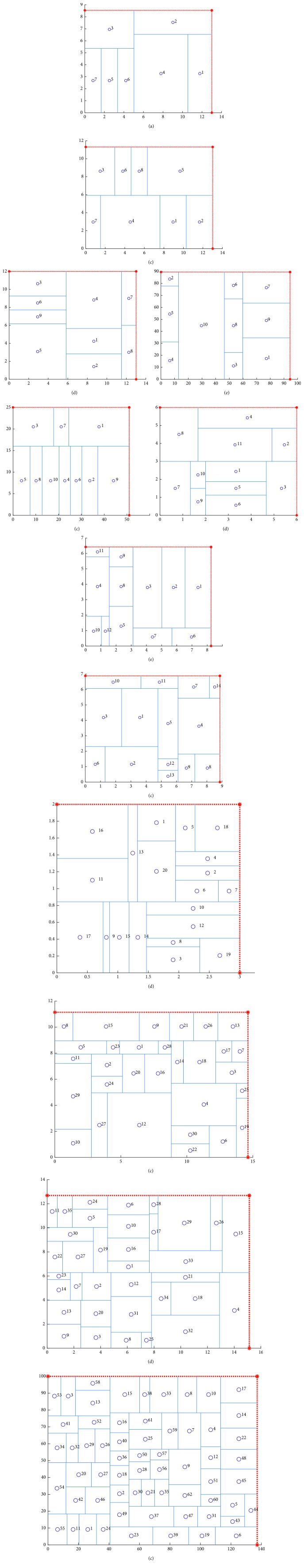
Optimal layout approach for the test cases. (a) Optimal layout approach for O7. (b) Optimal layout approach for O8. (c) Optimal layout approach for O9. (d) Optimal layout approach for F10. (e) Optimal layout approach for VC10. (f) Optimal layout approach for MB11. (g) Optimal layout approach for Ba12. (h) Optimal layout approach for Ba14. (i) Optimal layout approach for AB20. (j) Optimal layout approach for SC30. (k) Optimal layout approach for SC35. (l) Optimal layout approach for P62.

The optimal layout approach of all tested cases obtained by the proposed approach is presented in Figures 13(a)–13(l).

From Table 4, we found that the proposed approach can search better solutions than did the methods from literature for 8 of 12 tested problems. As shown, the proposed approach has more improvement when the facility number becomes bigger; for example, the improvement present can be up to 4–7% when the facility number reaches 30 and 35. Moreover, the average running time for finding the best solutions and the average total CPU running times have reduced drastically than did the approach of other literature. But, for the test case of P62 with no spare space for all the facilities, the proposed approach has a large gap from the optimal result of [35].

## 5. Conclusions and Prospects

Considering the constraints of area and aspect ratio of facilities, this paper proposed a slicing-tree-based binary plane segmentation method. The given area is divided into some blocks whose number equal to the number of facilities waiting for arrangement. The optimal solution was found by use of the layered coding genetic algorithm with the goal of maximizing the closeness relationship score and minimizing the material handling cost between facilities at the same time. The results of comparison of above 12 known problems between other literature methods and proposed approach show the effectiveness of the plane segmentation layout strategy and the reliability of layered coding genetic algorithm for solving the problems.

We draw the following conclusions based on the above study:

- It is reasonable and effective to partition the facilities layout area by use of the binary plane segmentation method, which can arrange a reasonable block area for each facility. So, the feasibility of solution was guaranteed during the iterative process of genetic algorithm.
- By use of the layered coding genetic algorithm, the optimal splitting approach, i.e., optimal facility layout scheme, will be found during multiple iterative process. The result can provide decision support for actual production facility layout.
- The plane splitting process was expressed well by the layered coding approach. When the UA-FLP problem changed, i.e., the facility number, facility area, and also the aspect ratio, these changed values can be assigned in the form of parameters; as a result, the corresponding optimal layout approach can be output quickly. Also, the layout approaches of different parameters can be compared easily in order to find the key influence factors.

The optimal layout approach of UA-FLP can be obtained fast by use of the plane segmentation method and layered coding genetic algorithm; the corresponding objective function value and the minimal area needed for locating facilities can also be provided. For multiproduct facility layout problem, different layout approaches for different products can be output by running the proposed algorithm in terms of the different requirement of product for logistics cost and closeness relationship between facilities, i.e., realizing a dynamic optimization for multiproduct facility layout. The facility number has a significant impact on performance of proposed algorithm, and there is the possibility of fall into local optimum, so the large-scale facility layout optimization algorithm and the combination scheme with the local search algorithm can be chosen as the direction of further research.

## Figures and Tables

**Algorithm 1:**
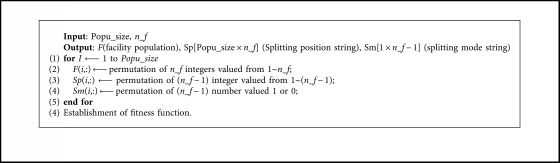
Coding of facility population, splitting position and splitting mode layer.

**Algorithm 2:**
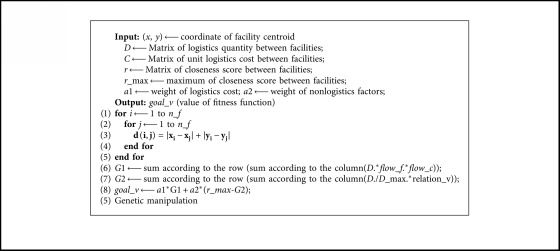
Calculating the value of fitness function.

**Algorithm 3:**
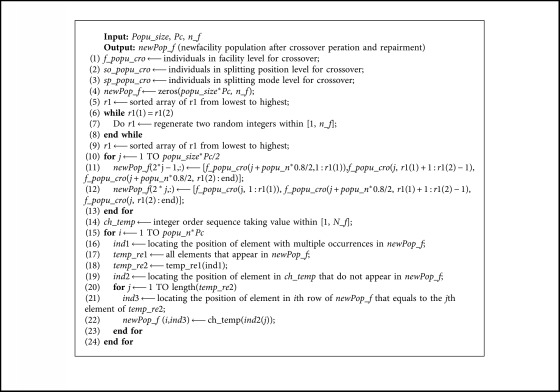
Crossover operation and repair in facility layer.

**Algorithm 4:**
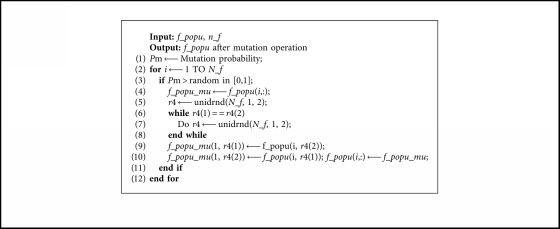
Mutation operation.

**Table 1:** Nonlogistics relationship level and corresponding quantitative score.

Level	*A*	*E*	I	*O*	*U*	*X*
Meaning	Absolutely necessary	Especially important	Important	Ordinary important	Unimportant	Closeness undesirable
Score	4	3	2	1	0	−1

**Table 2:** Results of comparison of tested problems between LCGA and other approach.

Problems	Facility number	Best known results of reference	Facility dimension [*W*, *H*]	Data reference
O7	7	134.19 [24]	[8.54, 13]	Meller et al. [28]
O8	8	245.51 [24]	[11.31, 13]	Meller et al. [28]
O9	9	241.06 [29]	[12, 13]	Meller et al. [28]
F10	10	8567.00 [30]	[90, 95]	Montreuil et al. [30]
VC10	10	22899.65 [24]	[51, 25]	Van Camp et al. [31]
MB11	11	1171 [32]	[6, 6]	Bozer et al. [33]
BA12	12	8021 [20]	[6, 10]	Bazaraa [34]
BA14	14	4665.93 [20]	[7, 9]	Bazaraa [34]
AB20-ar7	20	4793.47 [20]	[2, 3]	Armour and Buffa [1]
SC30	30	3563.95 [35]	[12, 15]	Liu and Meller [32]
SC35	35	3814.98 [35]	[15, 16]	Liu and Meller [32]
P62	62	3720521 [35]	[100, 137.18]	Komarudin and Wong [36]

**Table 3:** Results of comparison of tested problems between LCGA and other approach.

Problems	Population Size	Max generations	Crossover Probability	Mutation Probability	Selection method
O7	80	1000	0.7	0.05	Fitness-based
O8	80	1000	0.7	0.05	Fitness-based
O9	100	1000	0.7	0.05	Fitness-based
F10	100	1000	0.6	0.08	Fitness-based
VC10	100	1000	0.6	0.08	Fitness-based
MB11	120	1500	0.6	0.08	Fitness-based
BA12	120	1500	0.6	0.08	Fitness-based
BA14	200	2000	0.6	0.08	Fitness-based
AB20-ar7	300	2000	0.5	0.08	Fitness-based
SC30	500	2000	0.4	0.08	Fitness-based
SC35	500	2000	0.4	0.08	Fitness-based
P62	600	2000	0.3	0.1	Fitness-based

**Table 4:** Results of comparison of tested problems between LCGA and other approaches.

Problems	Best results of LCGA	Imp (%)	Average optimal iteration number	Average optimal searching time	Total CPU time (s)
O7	131.6773	−1.87	85	10.8	136.2
O8	245.5002	0.00	78	13.2	144.5
O9	241.0616	0.00	101	15.1	164
F10	8449.7	−1.37	152	29.5	177.4
VC10	22845	−0.24	196	37.6	180
MB11	1278.1	9.15	619	112.3	187.06
BA12	8040.8	0.25	210	38.4	190
BA14	4592.24	−1.58	235	80.1	356.1
AB20-ar7	4805.47	0.25	510	384.6	1543.2
SC30	3412.87	−4.24	780	398.7	1895.3
SC35	3519.9	−7.73	856	465.4	1931
P62	439080	18.02	1120	3178.6	7605.28

Note: Imp (%) = (the result of the proposed approach in this paper–the best solution found in the literature in Table 1)/(the best solution found in the literature in Table 1)^*∗*^100.

## Data Availability

The data used to support the findings of this study are available from the corresponding author upon request.

## References

[1] Armour G. C., Buffa E. S. (1963). A heuristic algorithm and simulation approach to relative location of facilities. *Management Science*.

[2] Tompkins J. A. (2010). *Facilities Planning*.

[3] Liu F., Dong M., Hou W. H., Zhai Y. (2007). Multi criteria evaluation for facility layout problems. *Journal of Shanghai Jiaotong University*.

[4] Wang M.-J., Hu M. H., Ku M.-Y. (2005). A solution to the unequal area facilities layout problem by genetic algorithm. *Computers in Industry*.

[5] Haktanirlar U., Kulturel K. B. S. (2012). An artificial immune system based algorithm to solve unequal area facility layout problem. *Expert Systems with Applications*.

[6] Wang T.-Y., Lin H.-C., Wu K.-B. (1998). An improved simulated annealing for facility layout problems in cellular manufacturing systems. *Computers & Industrial Engineering*.

[7] Mak K. L., Wong Y. S., Chan F. T. S. (1998). A genetic algorithm for facility layout problems. *Computer Integrated Manufacturing Systems*.

[8] Georgiadis M. C., Schilling G., Rotstein G. E., Macchietto S. (1999). A general mathematical programming approach for process plant layout. *Computers & Chemical Engineering*.

[9] Kumar K. R., Hadjinicola G. C., Lin T. I. (1995). A heuristic procedure for the single-row facility layout problem. *European Journal of Operational Research*.

[10] Taghavi A., Murat A. (2011). A heuristic procedure for the integrated facility layout design and flow assignment problem. *Computers & Industrial Engineering*.

[11] Kothari R., Ghosh D. (2013). Insertion based Lin-Kernighan heuristic for single row facility layout. *Computers & Operations Research*.

[12] Krishnakumar K., Melkote S. N. (2000). Machining fixture layout optimization using the genetic algorithm. *International Journal of Machine Tools and Manufacture*.

[13] Vallapuzha S., De Meter E. C., Choudhuri S., Khetan R. P. (2002). An investigation into the use of spatial coordinates for the genetic algorithm based solution of the fixture layout optimization problem. *International Journal of Machine Tools and Manufacture*.

[14] Samarghandi H., Taabayan P., Jahantigh F. F. (2010). A particle swarm optimization for the single row facility layout problem. *Computers & Industrial Engineering*.

[15] Önüt S., Tuzkaya U. R., Doğaç B. (2008). A particle swarm optimization algorithm for the multiple-level warehouse layout design problem. *Computers & Industrial Engineering*.

[16] Solimanpur M., Vrat P., Shankar R. (2004). Ant colony optimization algorithm to the inter-cell layout problem in cellular manufacturing. *European Journal of Operational Research*.

[17] Chen Y. H. (2013). A new data structure of solution representation in hybrid ant colony optimization for large dynamic facility layout problems. *International Journal of Production Economics*.

[18] Matai R. (2015). Solving multi objective facility layout problem by modified simulated annealing. *Applied Mathematics and Computation*.

[19] Şahin R. (2011). A simulated annealing algorithm for solving the bi-objective facility layout problem. *Expert Systems with Applications*.

[20] Kulturel-Konak S. (2012). A linear programming embedded probabilistic tabu search for the unequal-area facility layout problem with flexible bays. *European Journal of Operational Research*.

[21] Suo X. H., Liu Z. Q. (2007). Modeling and solution algorithms for facility layout of manufacturing systems. *Computer Integrated Manufacturing*.

[22] Ye M. J., Zhou G. G. (2005). The application of genetic algorithm in the Bi-criteria layout problem with aisles. *Journal of Systems Engineering—Theory & Practice*.

[23] Aiello G., La Scalia G., Enea M. (2012). A multi objective genetic algorithm for the facility layout problem based upon slicing structure encoding. *Expert Systems with Applications*.

[24] Palomo-Romero J. M., Salas-Morera L., García-Hernández L. (2017). An island model genetic algorithm for unequal area facility layout problems. *Expert Systems with Applications*.

[25] García-Hernández L., Arauzo-Azofra A., Salas-Morera L., Pierreval H., Corchado E. (2015). Facility layout design using a multi-objective interactive genetic algorithm to support the DM. *Expert Systems*.

[26] Paes F. G., Pessoa A. A., Vidal T. (2016). A hybrid genetic algorithm with decomposition phases for the unequal area facility layout problem. *European Journal of Operational Research*.

[27] Gonçalves J. F., Resende M. G. C. (2015). A biased random-key genetic algorithm for the unequal area facility layout problem. *European Journal of Operational Research*.

[28] Meller R. D., Narayanan V., Vance P. H. (1998). Optimal facility layout design. *Operations Research Letters*.

[29] Wong K. Y., Komarudin (2010). Solving facility layout problems using flexible bay structure representation and ant system algorithm. *Expert Systems with Applications*.

[30] Montreuil B., Ouazzani N., Brotherton E. Coupling zone-based layout optimization, ant colony system and domain knowledge.

[31] van Camp D. J., Carter M. W., Vannelli A. (1992). A nonlinear optimization approach for solving facility layout problems. *European Journal of Operational Research*.

[32] Liu Q., Meller R. D. (2007). A sequence-pair representation and MIP-model-based heuristic for the facility layout problem with rectangular departments. *IIE Transactions*.

[33] Bozer Y. A., Meller R. D. (1997). A reexamination of the distance-based facility layout problem. *IIE Transactions*.

[34] Bazaraa M. S. (1975). Computerized layout design: a branch and bound approach. *A I I E Transactions*.

[35] Kulturel-Konak S., Konak A. (2011). Unequal area flexible bay facility layout using ant colony optimisation. *International Journal of Production Research*.

[36] Komarudin, Wong K. Y. (2010). Applying ant system for solving unequal area facility layout problems. *European Journal of Operational Research*.

